# Metabolomics Profiling Discriminates Prostate Cancer From Benign Prostatic Hyperplasia Within the Prostate-Specific Antigen Gray Zone

**DOI:** 10.3389/fonc.2021.730638

**Published:** 2021-10-15

**Authors:** Bei Xu, Yan Chen, Xi Chen, Lingling Gan, Yamei Zhang, Jiafu Feng, Lin Yu

**Affiliations:** ^1^ Department of Clinical Laboratory, Mianyang Central Hospital, School of Medicine, University of Electronic Science and Technology of China, Mianyang, China; ^2^ Department of Clinical Pharmacy, Sichuan Cancer Hospital & Institute, Sichuan Cancer Center, School of Medicine, University of Electronic Science and Technology of China, Chengdu, China; ^3^ Department of Application Support Center, SCIEX Analytical Instrument Trading Co., Shanghai, China

**Keywords:** prostate cancer, prostate-specific antigen, untargeted metabolomics, lipid metabolism, candidate biomarkers

## Abstract

**Objective:**

Prostate cancer (PCa) is the second most common male malignancy globally. Prostate-specific antigen (PSA) is an important biomarker for PCa diagnosis. However, it is not accurate in the diagnostic gray zone of 4–10 ng/ml of PSA. In the current study, the performance of serum metabolomics profiling in discriminating PCa patients from benign prostatic hyperplasia (BPH) individuals with a PSA concentration in the range of 4–10 ng/ml was explored.

**Methods:**

A total of 220 individuals, including patients diagnosed with PCa and BPH within PSA levels in the range of 4–10 ng/ml and healthy controls, were enrolled in the study. Liquid chromatography coupled with tandem mass spectrometry (LC-MS/MS)-based non-targeted metabolomics method was utilized to characterize serum metabolic profiles of participants. Principal component analysis (PCA) and partial least squares discriminant analysis (PLS-DA) methods were used for multivariate analysis. Receiver operating characteristic (ROC) curve analysis was performed to explore the diagnostic value of candidate metabolites in differentiating PCa from BPH. Correlation analysis was conducted to explore the relationship between serum metabolites and common clinically used fasting lipid profiles.

**Results:**

Several differential metabolites were identified. The top enriched pathways in PCa subjects such as glycerophospholipid and glycerolipid metabolisms were associated with lipid metabolism. Lipids and lipid-like compounds were the predominant metabolites within the top 50 differential metabolites selected using fold-change threshold >1.5 or <2/3, variable importance in projection (VIP) > 1, and Student’s t-test threshold *p* < 0.05. Eighteen lipid or lipid-related metabolites were selected including 4-oxoretinol, anandamide, palmitic acid, glycerol 1-hexadecanoate, dl-dihydrosphingosine, 2-methoxy-6*Z*-hexadecenoic acid, 3-oxo-nonadecanoic acid, 2-hydroxy-nonadecanoic acid, *N*-palmitoyl glycine, 2-palmitoylglycerol, hexadecenal, d-erythro-sphingosine C-15, *N*-methyl arachidonoyl amine, 9-octadecenal, hexadecyl acetyl glycerol, 1-(9*Z*-pentadecenoyl)-2-eicosanoyl-glycero-3-phosphate, 3*Z*,6*Z*,9*Z*-octadecatriene, and glycidyl stearate. Selected metabolites effectively discriminated PCa from BPH when PSA levels were in the range of 4–10 ng/ml (area under the curve (AUC) > 0.80). Notably, the 18 identified metabolites were negatively corrected with total cholesterol (TC), low-density lipoprotein cholesterol (LDL-C), and Apo-B levels in PCa patients; and some were negatively correlated with high-density lipoprotein cholesterol (HDL-C) and Apo-A levels. However, the metabolites were not correlated with triglycerides (TG).

**Conclusion:**

The findings of the present study indicate that metabolic reprogramming, mainly lipid metabolism, is a key signature of PCa. The 18 lipid or lipid-associated metabolites identified in this study are potential diagnostic markers for differential diagnosis of PCa patients and BPH individuals within a PSA level in the gray zone of 4–10 ng/ml.

## Introduction

Globally, prostate cancer (PCa) is the second most prevalent male malignancy, accounting for more than 1 million new cases and more than 0.35 million deaths (1). Currently, diagnosis and localization of PCa are mainly based on digital rectal examination (DRE) and assessment of serum prostate-specific antigen (PSA) levels and final verification through transrectal ultrasound-guided prostate biopsy (TRUSPB) (2). PSA is an androgen-regulated serine protease produced and secreted by prostate epithelial cells, with approximate range of normal levels at 0–1.5 ng/ml (3). Elevated levels of PSA (>4 ng/ml) are associated with PCa (1). However, high PSA levels are also observed in individuals with benign prostatic hyperplasia (BPH), prostatitis, and prostate injury (4, 5). More evidence demonstrates that the positive predictive value of PSA is averagely 21% within the gray zone of 4–10 ng/ml (2), implying that the PSA approach has a poor specificity for PCa diagnosis. Invasive TRUSPB-based histological examination is currently the main diagnostic approach for PCa; however, it is not routinely recommended for patients due to the tedious procedure and associated significant discomfort and complications. Moreover, most individuals without PCa undergo unnecessary TRUSPB owing to the poor specificity of the PSA test ultimately developing complications (6, 7). Therefore, it is imperative to develop novel effective and minimally invasive detection biomarkers for accurate PCa screening, mainly at the gray zone 4–10 ng/ml.

Aberrant cancer metabolism is a newly recognized hallmark of various malignancies (8, 9). Studies have previously used metabolomics, which is the systematic study of the metabolites, to elucidate chemical fingerprints that specific cellular states and processes leave behind, thus providing in-depth understanding of health and disease. Metabolomics analysis of biological fluids is an effective and minimally invasive technology for disease monitoring, and a potential diagnostic non-invasive tool (8, 10). Several cancers including colorectal cancer (11), pancreatic cancer (12), and gastric cancer (13) exhibit significantly altered metabolite concentrations between cancer patients and healthy controls (HCs). Additionally, previous studies have explored the potential role of metabolites in urine, blood (serum or plasma), seminal fluid, or (tumor) tissues in PCa progression (1, 14, 15). Evidences show that healthy prostate cells favor citrate synthesis over citrate utilization and mainly rely on glucose oxidation to provide energy, thus resulting in citrate accumulation (15–17). Accumulation of zinc in benign prostate cells inhibits activity of m-aconitase (ACO), which catalyzes conversion of citrate to isocitrate in the tricarboxylic acid (TCA) cycle (16, 18). However, prostate cells have no capacity to accumulate zinc following neoplastic transformation. As a result, m-aconitase activity and citrate oxidation are restored, which are accompanied by subsequent decrease in citrate accumulation and increase in ATP production (18, 19). Increased lipid biosynthesis, which is important for cellular proliferation and intercellular signaling, is a classical metabolic reprogramming in malignant transformation. To achieve increased lipid metabolism, citrate is converted to acetyl-CoA (a precursor for lipogenesis and cholesterogenesis) in the cytosol (18). Recent findings show upregulation of expression of several key enzymes associated with cholesterol and fatty acid synthesis, which are regulated by androgen; and the enzymes exhibit increased activity in PCa cells (18). Studies on plasma metabolites in circulation in PCa are mainly focused on glycine, alanine, sarcosine, and a few lipid metabolites (1, 14, 15). However, these biomolecules cannot discriminate patients with PCa and BPH in the PSA gray zone of 4–10 ng/ml. Moreover, the results on metabolites are not consistent across different studies. The differences are attributed to application of different methods for metabolomics analysis, differences in experimental designs, and varying characteristics of the study participants (20). Metabolomics is a rapidly growing new field with significant methodological and technical hurdles; therefore, more definitive studies focusing on metabolic pathways potentially altered during prostate tumorigenesis and progression should be conducted.

**Figure 1:**
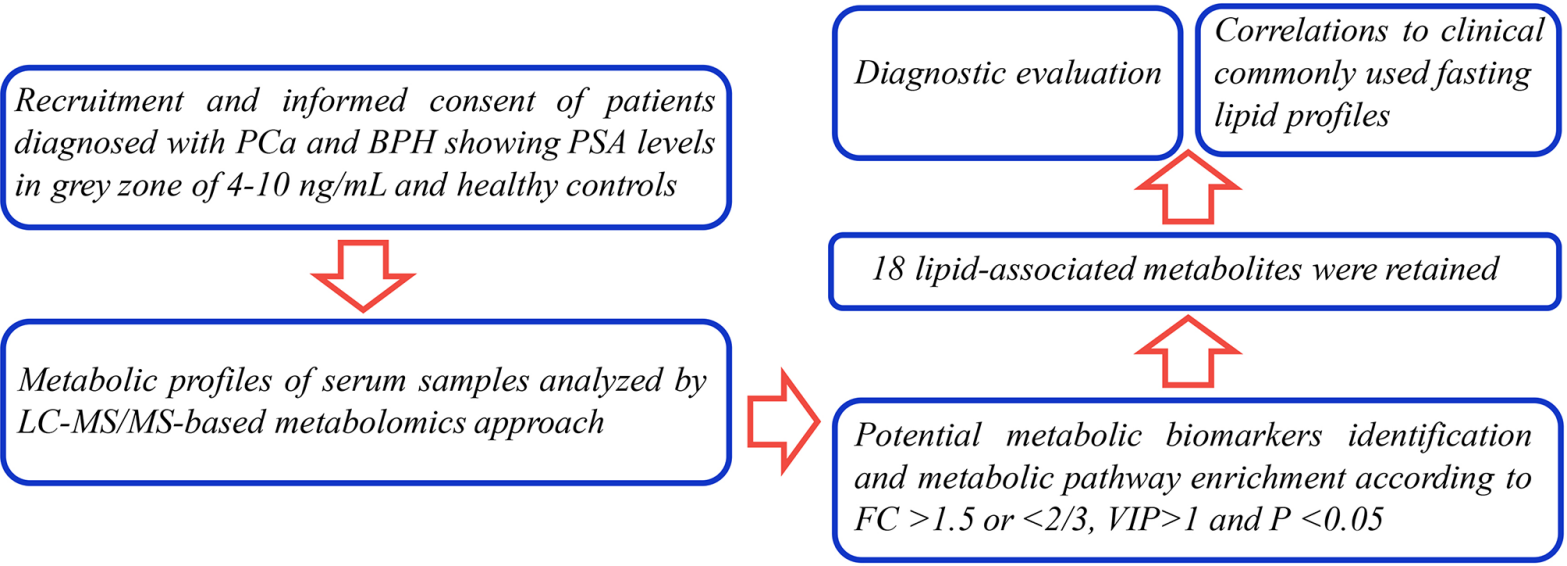
The workflow of the analysis steps.

The currents study sought to explore novel potential serum metabolic biomarkers in PCa patients within the gray zone of 4–10 ng/ml through liquid chromatography coupled with tandem mass spectrometry (LC-MS/MS) analysis. Previous studies mainly focused on alterations of metabolites in PCa patients in high PSA levels (always >10 ng/ml) compared with BPH individuals. In the present study, levels of novel metabolic candidates in PCa patients were compared with the levels in BPH controls within the gray zone of 4–10 ng/ml. The findings of the current study showed that the identified metabolites effectively distinguished two common disease conditions with PSA levels in the clinical diagnostic gray zone, therefore, have a high potential for clinical decision making (**Figure 1**).

## Materials and Methods

### Study Design and Participants

A total of 74 PCa patients, 74 BPH patients, and 72 healthy subjects were enrolled in the current prospective pilot study. The study protocol was approved by the Medical Ethics Committee of Mianyang Central Hospital. Patients were diagnosed with PCa through TRUSPB method but had not undergone any treatment. Men diagnosed with BPH were used as the disease control group. Hematoxylin and eosin (H&E) staining and immunohistochemical assay of alpha-methylacyl-CoA racemase (AMACR, P504S), high-molecular-weight cytokeratin (HMWCK), and P63 antigens were done on the prostate specimens. Briefly, specimens were formalin-fixed and embedded in paraffin. Serial sections of 5-μm thickness were cut and stained with H&E. Sections were processed for immunohistochemistry, as follows. Sections were dewaxed in xylene and hydrated in graded ethanol and water. Antigen retrieval was obtained by boiling in 0.01 mol/L of citrate buffer (pH 6.0) for 20 min. Sections were then respectively incubated with the primary antibodies at a dilution of 1:100 for 30 min at room temperature. Immunostaining was performed with a streptavidin-biotin complex kit (Beyotime Biotechnology Co. Ltd, Shanghai, China). After incubation with diaminobenzidine (DAB) as a chromogen, the specimens were counterstained with Harris hematoxylin and cover slipped. Staining was observed using the light microscope at ×200 magnification (Olympus, Tokyo, Japan). The represent pathological section staining is shown in **Supplementary Figure S1**.

Participant characteristics are presented in **Table 1**. The range of serum PSA concentrations of patients with PCa or BPH was 4–10 ng/ml. Analysis showed no significant differences among the three groups except for PSA (referring to total PSA (TPSA) in this study) and Gleason scores. PCa and BPH groups showed significantly higher prostate PSA levels (both *p* < 0.001) compared with the levels in the HC group. Notably, PSA levels in the PCa and BPH groups were not significantly different. The number of PCa patients with GS4–GS9 was 18, 25, 17, 9, 3, and 2, respectively.

**Table 1:** Characteristics of the individuals included in this study.

	HC group (n = 72)	BPH group (n = 74)	PCa group (n = 74)
Age (years)	62.1 ± 8.7	62.2 ± 12.2	64.1 ± 8.2
BMI (kg/m^2^)	27.1 ± 6.1	26.1 ± 5.7	26.4 ± 6.4
Prostate volume (ml)	47.3 ± 26.6	52.6 ± 18.9	46.9 ± 25.7
FPSA (ng/ml)	0.66 ± 0.51	1.19 ± 0.73	1.06 ± 0.41
TPSA (ng/ml)	2.24 ± 1.09	7.68 ± 2.81^###^	8.69 ± 2.16^***^
Tumor Gleason score	NA	NA	5 (4–9)

BMI, body mass index; FPSA, free prostate-specific antigen; TPSA, total prostate-specific antigen; HC, healthy control; BPH, benign prostatic hyperplasia; PCa, prostate cancer.

BPH group compared with HC group, ^###^ *p* < 0.001; PCa group compared with HC group, ^***^ *p* < 0.001.

### Sample Collection and Analysis

Venous blood was collected from all participants between 6:00 and 10:00 a.m. after undergoing overnight fasting to eliminate diet-induced variations. Serum was obtained from blood samples by centrifugation (3,000 rpm for 15 min). All clinical laboratory tests were performed within 2 h after serum collection and were stored at −80°C for subsequent analysis.

Concentrations of triglycerides (TG), total cholesterol (TC), high-density lipoprotein cholesterol (HDL-C), and low-density lipoprotein cholesterol (LDL-C) were evaluated using the same serum sample through LC-MS/MS analysis.

### Sample Preparation for Liquid Chromatography–MS/MS Analysis

A mixture of serum samples (190 μl), internal standard (10 μl) (a mixture of clenbuterol and chloramphenicol for the positive and negative ion modes, respectively), and methanol–acetonitrile (1:1 v/v) (800 μl) was incubated at −20°C for 1 h. The mixture was then vortexed and centrifuged, and the supernatant was obtained as previously described (13). The resulting solutions were filtered through a 0.22-µm microporous membrane. A 5 µl aliquot of the filtrate was transferred into an autosampler and injected into the LC-MS/MS system for metabolomics analysis. Aliquots of all serum samples (10 µl) were pooled to prepare quality control (QC) samples. Pretreatment of the QC samples was performed following the same procedure as that of the study samples. QC samples were introduced every 10 samples in the analytical sequence to evaluate the reliability of the large-scale metabolomics analysis (21).

### Instrumentation and Liquid Chromatography–MS/MS Conditions

Analysis was performed using an Agilent^®^1290 Infinity II UPLC system (Agilent Technologies Inc., USA) coupled to a Triple TOF 5600+ mass spectrometer system (AB Sciex, Framingham, MA). A Waters ACQUITY HSS T3 C18 column (100 × 2.1 mm, i.d. 1.8 µm) was used for separation of components. Continuous evaluation of the full-scan survey MS data was performed by an Acquisition software (Analyst TF1.7, AB Sciex) using preselected criteria for acquisition of MS/MS spectra. The mobile phase comprised water with 0.1% (v/v) formic acid (solvent A) and acetonitrile with 0.1% (v/v) formic acid (solvent B). The gradient for the mobile phase was as follows: a linear gradient of 99% A over initial–1.2 min; 99%–30% A over 1.2–4.5 min; 30%–1% A over 4.5–13.0 min; 1% A over 13.0–16.5 min; 1%–99% A over 16.5–16.6 min; and 99% A over 16.6–20.0 min. The flow rate was set at 0.30 ml/min. The column temperature was set as 30°C. An electrospray ionization (ESI) source was used with positive and negative modes for the mass spectrometer system. Electrospray source parameters were set as follows: curtain gas (CUR), 35 psi; ion source gas 1 (GS1), 55 psi; ion source gas2 (GS2), 55 psi; temperature (TEM), 550°C; declustering potential (DP), ± 80 V; collision energy (CE), ± 10 V; and accumulation time (AT), 0.15 s.

### Identification of Differential Metabolites and Metabolic Pathway Analysis

Analyst TF (version 1.7.1, AB Sciex, USA) qualitative analysis software was used for acquisition and processing of the raw data. A metabolomics data processing workflow was then established with a serial set of processes including peak picking, quality assurance, normalization, missing value imputation, transformation, and scaling. The processed molecular weights of the metabolites (molecular weight error < 20 ppm) were confirmed, matched, and annotated using a standard database, custom databases (Metlin, MassBank, LipidMaps, Mzclound, HMDB, and ONE-MAP databases), and other integrated databases to achieve accurate metabolite characterization.

Metabolites with fold-change threshold >1.5 or <2/3, variable importance in projection (VIP) >1, and Student’s t-test threshold *p* < 0.05 were selected as differential metabolites for group discrimination. Metabolic pathways associated with differential metabolites were explored using Kyoto Encyclopedia of Genes and Genomes (KEGG) and MetaboAnalyst databases through comparison between the change in metabolite ion intensity and the corresponding controls.

The metabolomics community proposed defined metrics for assessing the confidence of an annotation (22). All metabolites identified in the current study were defined “annotation” (levels 2) and did not require exhaustive analytical validation. This was according to formal definitions of metabolite annotation and identification developed by the Chemical Analysis Working Group of the Metabolomics Standards Initiative (MSI) (23).

### Statistical Analysis

All statistical analyses were performed with SPSS 25.0 software (International Business Machines Corp., USA). Normally distributed continuous data were expressed as mean ± standard deviation (SD), and comparisons between two groups were performed using Student’s t-test. Non-normally distributed variables were expressed as the median and interquartile range (IQR) and compared using the Mann–Whitney U tests. Differences among multiple groups were compared using one-way ANOVA if the variances were equal. Welch’s approximate analysis of variance was applied followed by Dunnett’s T3 test when the variances were uneven. Pearson’s or Spearman’s bivariate correlation analysis were performed for normal or skewed distribution to explore the relationship between selected metabolites parameters and commonly used fasting lipid profiles. A *p*-value less than 0.05 denoted statistical significance.

Multivariate analysis was performed using SIMCA 15.0.2 software (Umetrics AB, Umea, Sweden). LC-MS/MS data were subjected to principal component analysis (PCA) using an unsupervised non-targeted approach, to visualize metabolome variation among groups. Metabolome differences between sample pairs were maximized using a supervised classification method with unit variance scaling of partial least squares discriminant analysis (PLS-DA), and key variables contributing to the classification were identified according to their VIP (24). Permutation tests with 200 random variables were conducted to avoid data overfitting in the PLS-DA model. Diagnostic performance of differential metabolites was evaluated using receiver operating characteristic (ROC) curves. The area under the curve (AUC) was recorded as a measure of diagnostic accuracy and compared among group. The trade-offs between sensitivity and specificity for each variable were aggregated. AUC of 1.0 denoted 100% sensitivity and specificity, indicating perfect assignment, whereas an AUC of 0.5 indicated an unreliable test (gray line) (25). SPSS 25.0 (International Business Machines Corp., USA) was used for correlation analysis between metabolites and clinical parameters.

## Results

### Multivariate Statistical Analysis of Metabolites

LC-MS/MS is an important technique that generates two-dimensional profiles of constituent compounds over retention time (RT) and uses mass-to-charge ratio (m/z) for metabolomics analysis of biological samples. LC-MS/MS method was used for analysis of 220 serum samples and 20 QC samples in the positive (ESI+) and negative (ESI−) ion modes. Processed data comprising RT, exact mass, and peak intensity were subjected to multivariate statistical analysis.

**Figure 2:**
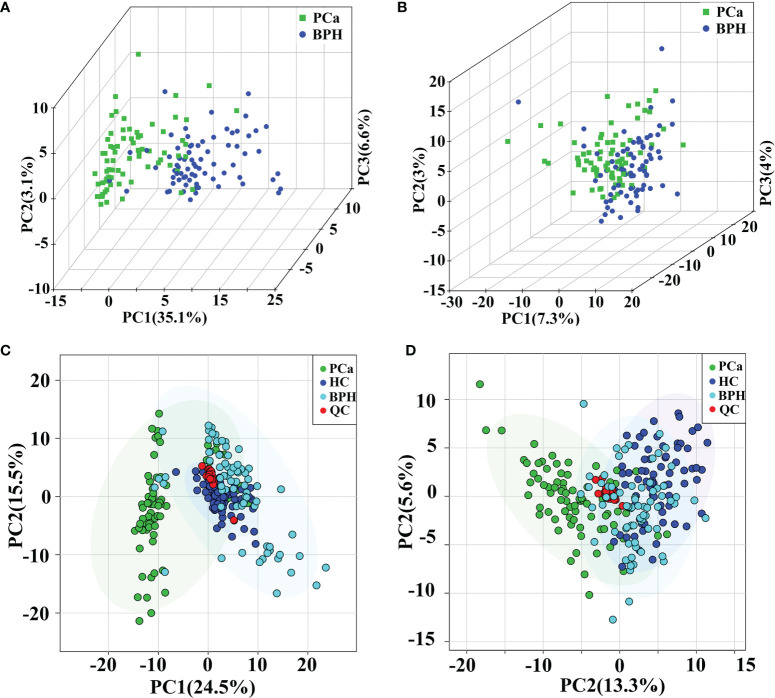
PCA score plots in positive **(A)** and negative **(B)** ion modes between PCa and BPH groups. PCA score plots of QC samples in positive **(C)** and negative **(D)** ion modes among PCa, BPH, and HC groups. PCA, principal component analysis; PCa, prostate cancer; BPH, benign prostatic hyperplasia; QC, quality control; HC, healthy control.

General clusters, trends, or outliers among the observations were visualized using PCA. Score plots demonstrated a direct image of observational distributions. A distinct classification was achieved for the PCa, BPH, and HC groups in positive ion mode (**Figures 2A, C**). The results showed effective separation of the principal components of PCa, BPH, and HC groups in positive ion mode. However, PCa, BPH, and HC groups were not effectively clustered under the negative ion mode (**Figures 2B, D**); therefore, multivariate statistical analysis was needed to further explore their relationship. The pooled QC samples clustered together in the PCA plots in both positive and negative ion modes (**Figures 2C, D**, and **Supplementary Figure S2**), indicating stability and repeatability of the analysis system.

**Figure 3:**
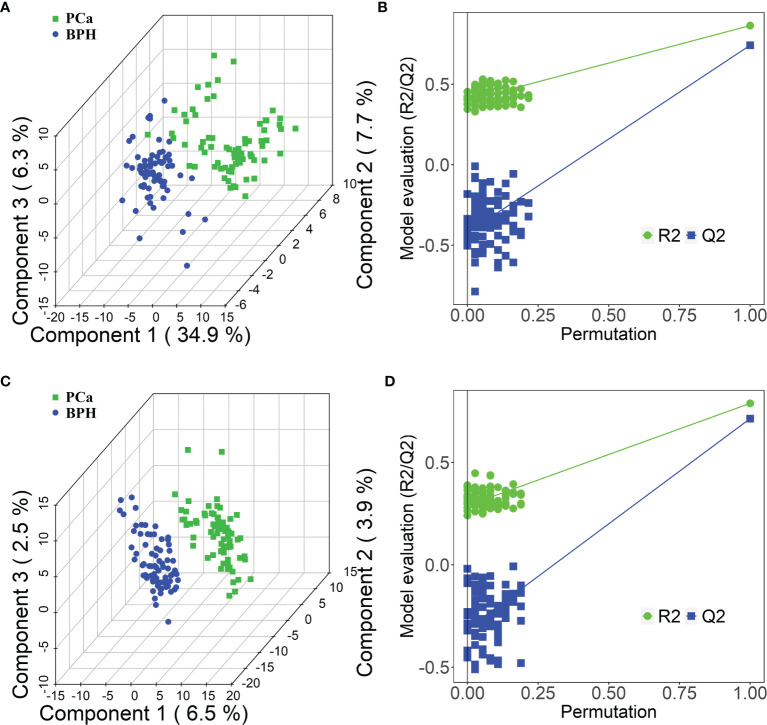
PLS-DA score plots in positive **(A)** and negative **(C)** ion modes between PCa and BPH groups. PLS-DA permutation test plots in positive **(B)** and negative **(D)** ion modes between PCa and BPH groups. The criterion for evaluating whether there is overfitting in the PLS-DA model is that the regression line at a blue Q2 point crosses or is less than 0 from the abscissa. PLS-DA, partial least squares discriminant analysis; PCa, prostate cancer; BPH, benign prostatic hyperplasia.

Moreover, PLS-DA was performed to further explore the different metabolic profiles, as the method has a better discriminative power compared with that of PCA and to identify potential biomarkers. Distinct clustering was observed for the PCa and BPH groups in both positive (**Figure 3A**) and negative (**Figure 3C**) ion modes, indicating accurate separation of the two groups. The R^2^X, R^2^Y, and Q^2^ (cum) parameters in PLS-DA method were mainly applied for model evaluation. The parameters of modeling, R^2^X, R^2^Y, and Q^2^ (cum) in the positive ion mode were established as 0.23, 0.80, and 0.72, respectively. The R^2^X, R^2^Y, and Q^2^ (cum) for the negative mode were 0.36, 0.82, and 0.75. High values of Q^2^ indicated high accuracy of the PLS-DA model. A 200 times’ random permutation test was performed to explore the overfitting of the supervised PLS-DA models. The Y-intercepts of Q^2^ distributions were less than zero in both positive and negative ion modes (**Figures 3B, D**), indicating reliability of the established PLS-DA model. Moreover, distinct clustering of the HC and PCa or BPH groups in both positive and negative ion modes was observed (**Supplementary Figures S3A**–**H**). In summary, PCA and PLS-DA models indicated significant distinction among PCa, BPH, and HC groups and were highly effective in characterizing serum metabolites.

### Identification of Potential Metabolites and Pathways

**Figure 4:**
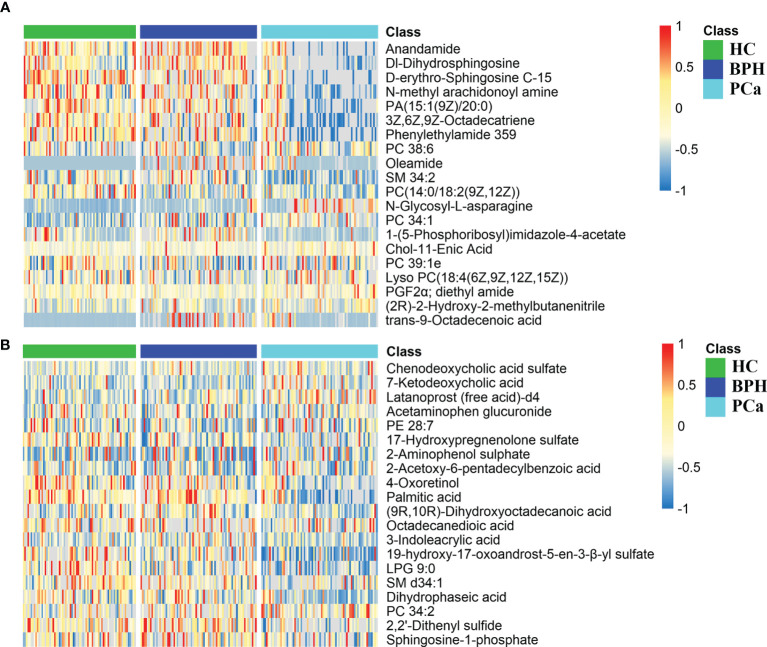
Differential metabolite heat maps in positive **(A)** and negative **(B)** modes. The columns represent samples, the rows represent metabolites, and the relative content of the metabolites is displayed by color. The heat map shows differential metabolites among HC, BPH, and PCa groups. HC, healthy control; BPH, benign prostatic hyperplasia; PCa, prostate cancer.

Peaks were aligned, and missing values were eliminated from the MS/MS data, resulting in 6,891 and 7,868 peaks in the ESI+ and ESI− modes, respectively. Qualitative identification was conducted using publicly available databases, standard compounds databases, and several integrated databases. A total of 1,755 metabolites were identified in the positive ion mode, and 963 metabolites were identified in the negative ion mode. Subsequently, 362 different metabolites were selected using a fold-change threshold >1.5 or <2/3, VIP > 1, and Student’s t-test threshold *p* < 0.05. Heat maps of the 20 differential metabolites detected in the positive and negative modes showed distinct clustering for each group, which was consistent with the orthogonal PLS-DA (OPLS-DA) results (**Figures 4A, B**). The most abundant class of metabolites was lipids and lipid-like molecules, including anandamide, dl-dihydrosphingosine, 4-oxoretinol, and palmitic acid.

In addition, the differential metabolites were enriched in metabolic pathways. Pathway impact values were acquired as cumulative percentage of the matched metabolite nodes. *p*-Values were calculated by enrichment analysis based on false discovery rate (FDR) [−log(*p*) value]. Results of metabolic pathway analysis showed that the differential metabolites in PCa were significantly mainly enriched in glycerophospholipid metabolism, glycerolipid metabolism, arachidonic acid metabolism, nicotinate and nicotinamide metabolism, and biotin metabolism pathways compared with those in the BPH group. Notably, glycerophospholipid metabolism was the top significantly enriched pathway in PCa at the gray zone of 4–10 ng/ml. Moreover, lipid metabolism-related pathways were the most significantly enriched metabolic pathways. Detailed information on the pathways is presented in **Table 2** and **Supplementary Table S1**. Significantly enriched metabolic pathways and the related metabolites between PCa and HC groups are presented in **Supplementary Table S2**.

**Table 2:** Significantly altered metabolic pathways between PCa and BPH groups with PSA levels at gray zone of 4–10 ng/ml.

Pathway name	KEGG.id	−log(*p*)	Impact	Hits
Glycerophospholipid metabolism	hsa00564	5.18	0.42	6
Glycerolipid metabolism	hsa00561	3.32	0.22	3
Arachidonic acid metabolism	hsa00590	0.67	0.31	2
Nicotinate and nicotinamide metabolism	hsa00760	0.66	0.19	1
Biotin metabolism	hsa00780	0.96	0.15	1

Impact, impact value of metabolic pathway determined by topology analysis; Hits, the number of differential metabolites matching the pathway; PCa, prostate cancer; BPH, benign prostatic hyperplasia; PSA, prostate-specific antigen; KEGG, Kyoto Encyclopedia of Genes and Genomes.

### Receiver Operating Characteristic Analysis of Selected Metabolites Discriminating Prostate Cancer Patients From Benign Prostatic Hyperplasia Patients

**Figure 5:**
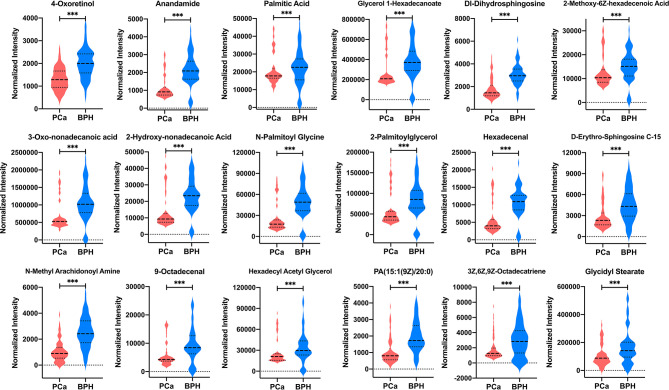
The normalized intensity peak areas of 18 differential metabolites in PCa and BPH groups. ^***^ *p* < 0.001. PCa, prostate cancer; BPH, benign prostatic hyperplasia.

A total of 18 lipid or lipid-like metabolites (4-oxoretinol, anandamide, palmitic acid, glycerol 1-hexadecanoate, dl-dihydrosphingosine, 2-methoxy-6*Z*-hexadecenoic acid, 3-oxo-nonadecanoic acid, 2-hydroxy-nonadecanoic acid, *N*-palmitoyl glycine, 2-palmitoylglycerol, hexadecenal, d-erythro-sphingosine C-15, *N*-methyl arachidonoyl amine, 9-octadecenal, hexadecyl acetyl glycerol, 1-(9*Z*-pentadecenoyl)-2-eicosanoyl-glycero-3-phosphate [PA (15:1(9*Z*)/20:0)], 3*Z*,6*Z*,9*Z*-octadecatriene, and glycidyl stearate) were selected as candidate biomarkers for early diagnosis of PCa at the gray zone of 4–10 ng/ml using the following criteria: variable VIP > 2, fold change (FC) >1.5 or <2/3, and *p* < 0.05 (**Table 3**). The normalized intensity peak areas of the selected metabolites are presented in **Figure 5**. The levels of the 18 metabolites were significantly lower in PCa patients compared with the corresponding levels in BPH (all *p* < 0.001).

**Table 3:** Detection of 18 selected lipid metabolites as potential biomarkers for the diagnosis of PCa with PSA levels at gray zone of 4–10 ng/ml.

Biomarker	FC	VIP	*p*-Value	AUC (95% CI)	Se (%)	Sp (%)	Trend (cancer)
4-Oxoretinol	0.65	2.71	6.51E−12	0.811 (0.738–0.870)	71.62	77.03	↓^***^
Anandamide	0.31	2.65	2.27E−22	0.890 (0.828–0.935)	85.14	90.54	↓^***^
Palmitic acid	0.28	2.59	5.88E−21	0.878 (0.814–0.926)	79.73	91.89	↓^***^
Glycerol 1-hexadecanoate	0.22	2.56	3.43E−20	0.873 (0.808–0.922)	77.03	93.24	↓^***^
dl-Dihydrosphingosine	0.59	2.22	5.25E−14	0.838 (0.769–0.893)	74.32	93.24	↓^***^
2-Methoxy-6*Z*-hexadecenoic acid	0.25	2.67	1.02E−22	0.889 (0.827–0.935)	82.43	91.89	↓^***^
3-Oxo-nonadecanoic acid	0.20	2.63	9.70E−22	0.881 (0.818–0.928)	81.08	90.54	↓^***^
2-Hydroxy-nonadecanoic acid	0.24	2.61	2.98E−21	0.884 (0.821–0.931)	81.08	91.89	↓^***^
*N*-Palmitoyl glycine	0.23	2.58	1.60E−20	0.862 (0.795–0.913)	81.08	90.54	↓^***^
2-Palmitoylglycerol	0.22	2.55	1.06E−19	0.848 (0.780–0.902)	79.73	90.54	↓^***^
Hexadecenal	0.32	2.51	4.03E−19	0.841 (0.772–0.896)	79.73	90.54	↓^***^
d-Erythro-sphingosine C-15	0.33	2.37	5.44E−17	0.871 (0.807–0.921)	77.03	90.54	↓^***^
*N*-Methyl arachidonoyl amine	0.42	2.30	1.68E−15	0.853 (0.785–0.906)	85.14	75.68	↓^***^
9-Octadecenal	0.23	2.27	6.16E−15	0.853 (0.786–0.906)	74.32	90.54	↓^***^
Hexadecyl acetyl glycerol	0.25	2.24	5.78E−14	0.833 (0.763–0.889)	78.38	89.19	↓^***^
PA(15:1(9*Z*)/20:0)	0.44	2.14	4.89E−13	0.839 (0.769–0.894)	75.68	89.19	↓^***^
3*Z*,6*Z*,9*Z*-Octadecatriene	0.27	2.07	2.24E−11	0.800 (0.726–0.861)	81.08	77.03	↓^***^
Glycidyl stearate	0.22	2.05	1.88E−11	0.804 (0.731–0.865)	79.73	79.73	↓^***^

Se, sensitivity; Sp, specificity; PCa, prostate cancer; PSA, prostate-specific antigen; FC, fold change; VIP, variable importance in projection; AUC, area under the curve; BPH, benign prostatic hyperplasia.

PCa group compared with BPH group, ^***^ *p* < 0.001.

**Figure 6:**
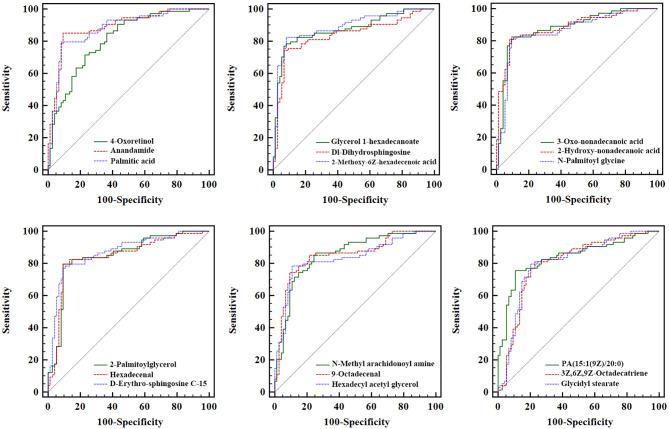
Receiver operating characteristic analysis of the 18 newly found candidate biomarkers for separating PCa from BPH. PCa, prostate cancer; BPH, benign prostatic hyperplasia.

ROC analysis of each biomarker was performed to their predictive value in PCa (**Table 3** and **Figure 6**). The results indicated that the 18 metabolites effectively discriminated PCa from BPH (AUC > 0.80).

### Correlations Between Differential Metabolites and Serum Fasting Lipid Profiles

Levels of TC, HDL-C, LDL-C, and Apo-A1 were statistically significantly different among HC, BPH, and PCa groups (all *p* < 0.05) (**Table 4**). Further analysis indicated that the levels of commonly used fasting lipid profiles including TC, TG, HDL-C, LDL-C, and Apo-A1 (except Apo-B) were significantly different in PCa group compared with the levels in HC subjects. These findings indicated a potential association between lipid metabolism and occurrence and progression of PCa. Notably, differences in the levels of these clinical lipid parameters were not statistically significant between HC and BPH groups, implying that these profiles are not involved in prostatitis. Moreover, significantly higher serum concentrations of HDL-C and Apo-A1 were observed in PCa patients compared with the levels in BPH patients. These findings indicated significant changes in lipid metabolism in cancer patients.

**Table 4:** Serum lipids and apolipoproteins profile for participants.

Fasting lipid profiles	HC (n = 72)	BPH (n = 74)	PCa (n = 74)	F, *p*
TC (mmol/L)	4.97 ± 0.91	4.95 ± 0.99	5.38 ± 1.48^***^	9.06, <0.001
TG (mmol/L)	1.77 ± 1.40	1.87 ± 1.49	1.53 ± 1.02^*^	2.37, 0.941
HDL-C (mmol/L)	1.42 ± 0.32	1.33 ± 0.26	1.49 ± 0.32^*,##^	5.49, 0.004
LDL-C (mmol/L)	3.04 ± 0.79	2.99 ± 0.72	3.29 ± 1.27^**^	4.60, 0.010
Apo-A1 (g/L)	1.48 ± 0.23	1.42 ± 0.22	1.56 ± 0.25^**,###^	8.54, <0.001
Apo-B (g/L)	1.00 ± 0.24	0.98 ± 0.22	1.04 ± 0.31	1.30, 0.272

HC, healthy control; BPH, benign prostatic hyperplasia; PCa, prostate cancer; TC, total cholesterol; TG, triglycerides; HDL-C, high-density lipoprotein cholesterol; LDL-C, low-density lipoprotein cholesterol.

Compared with healthy controls, ^***^ *p* < 0.001, ^**^ *p* < 0.01, ^*^ *p* < 0.05.

Compared with BPH subjects, ^###^ *p* < 0.001, ^##^ *p* < 0.01.

Furthermore, correlation analysis was performed to explore the relationship between the 18 lipid or lipid-like metabolites and the commonly used fasting lipid profiles. All the 18 lipid-related metabolites were significantly negatively correlated with TC, LDL-C, and Apo-B (all *p* < 0.05) (**Table 5**). Out of the 18 metabolites, four (4-oxoretinol, anandamide, palmitic acid, and glycerol 1-hexadecanoate) were negatively associated with HDL-C (all *p* < 0.05), whereas the other metabolites, including dl-dihydrosphingosine, 2-methoxy-6*Z*-hexadecenoic acid, and *N*-palmitoyl glycine, had no correlation with it. Moreover, Apo-A1 exhibited a negative correlation with lipid profiles such as 4-oxoretinol, palmitic acid, and glycerol 1-hexadecanoate but was not correlated with anandamide, dl-dihydrosphingosine, 9-octadecenal, 3*Z*,6*Z*,9*Z*-octadecatriene, and glycidyl stearate. Notably, TG was not correlated with all the 18 candidate biomarkers (all *p* > 0.05), implying that the newly identified lipid biomarkers are not involved in the TG metabolism pathway.

**Table 5:** Correlation analysis of differential metabolites and commonly used fasting lipid profiles.

Biomarker	TC (r, *p*)	TG (r, *p*)	HDL-C (r, *p*)	LDL-C (r, *p*)	Apo-A1 (r, *p*)	Apo-B (r, *p*)
4-Oxoretinol	−0.578, <0.001	0.031, 0.794	−0.242, 0.038	−0.502, <0.001	−0.241, 0.038	−0.313, 0.007
Anandamide	−0.542, <0.001	0.033, 0.782	−0.245, 0.035	−0.571, <0.001	−0.214, 0.067	−0.376, 0.001
Palmitic acid	−0.561, <0.001	−0.094, 0.426	−0.255, 0.028	−0.544, <0.001	−0.294, 0.011	−0.481, <0.001
Glycerol 1-hexadecanoate	−0.591, <0.001	−0.027, 0.822	−0.295, 0.011	−0.440, <0.001	−0.354, 0.002	−0.285, 0.014
dl-Dihydrosphingosine	−0.283, 0.015	−0.057, 0.631	−0.076, 0.519	−0.412, <0.001	−0.083, 0.483	−0.429, <0.001
2-Methoxy-6*Z*-hexadecenoic acid	−0.587, <0.001	−0.079, 0.504	−0.215, 0.066	−0.527, <0.001	−0.295, 0.011	−0.424, <0.001
3-Oxo-nonadecanoic acid	−0.582, <0.001	−0.081, 0.491	−0.246, 0.035	−0.463, <0.001	−0.329, 0.004	−0.324, 0.005
2-Hydroxy-nonadecanoic acid	−0.566, <0.001	−0.092, 0.436	−0.269, 0.020	−0.435, <0.001	−0.359, 0.002	−0.280, 0.016
*N*-Palmitoyl glycine	−0.524, <0.001	−0.076, 0.521	−0.223, 0.056	−0.459, <0.001	−0.240, 0.039	−0.314, 0.007
2-Palmitoylglycerol	−0.483, <0.001	−0.174, 0.139	−0.191, 0.103	−0.459, <0.001	−0.277, 0.017	−0.360, 0.002
Hexadecenal	−0.576, <0.001	−0.124, 0.291	−0.272, 0.019	−0.461, <0.001	−0.319, 0.006	−0.304, 0.008
d-Erythro-sphingosine C-15	−0.569, <0.001	−0.122, 0.299	−0.225, 0.054	−0.445, <0.001	−0.266, 0.022	−0.361, 0.002
*N*-Methyl arachidonoyl amine	−0.552, <0.001	−0.055, 0.641	−0.242, 0.038	−0.475, <0.001	−0.281, 0.015	−0.279, 0.016
9-Octadecenal	−0.461, <0.001	−0.150, 0.203	−0.063, 0.596	−0.489, <0.001	−0.111, 0.347	−0.445, <0.001
Hexadecyl acetyl glycerol	−0.474, <0.001	−0.038, 0.746	−0.242, 0.038	−0.426, <0.001	−0.280, 0.016	−0.326, 0.005
PA (15:1(9*Z*)/20:0)	−0.549, <0.001	0.067, 0.570	−0.283, 0.015	−0.458, <0.001	−0.231, 0.047	−0.305, 0.008
3*Z*,6*Z*,9*Z*-Octadecatriene	−0.416, <0.001	−0.024, 0.839	−0.075, 0.525	−0.409, <0.001	−0.069, 0.561	−0.360, 0.002
Glycidyl stearate	−0.463, <0.001	−0.152, 0.196	−0.066, 0.575	−0.484, <0.001	−0.124, 0.292	−0.484, <0.001

TC, total cholesterol; TG, triglycerides; HDL-C, high-density lipoprotein cholesterol; LDL-C, low-density lipoprotein cholesterol.

## Discussion

Patients classified in the PSA level arrangement gray zone with moderate Gleason sum scores are characterized by low or moderate risk. Therefore, their therapeutics must be tailored ideally according to the tumor characteristics (14). In this condition, “active surveillance,” which is increasingly recognized as initial treatment approach for men with low-risk PCa, is introduced (26, 27). However, repeated PSA assessments coupled with re-biopsies are required, which are associated with additional costs and risks. Currently, several biomarkers are available for early diagnosis of PCa (28); however, they are not effective for discriminating patients with PCa and BPH in the PSA gray zone of 4–10 ng/ml. Therefore, the present study sought to determine serum metabolic profiles between PCa and BPH patients with PSA levels at 4–10 ng/ml, for use as potential non-invasive candidate biomarkers for accurate PCa diagnosis.

Dependence of androgens is an important hallmark of PCa. Androgens are implicated in regulation of lipid metabolism through the sterol regulatory element-binding proteins (SREBPs) associated pathway (29). Dysregulation of lipid metabolism affects several cellular processes such as proliferation, differentiation, and motility, which are highly involved in carcinogenesis pathways (such as transformation, progression, and metastasis) (30, 31). In the current study, dysregulation of lipid metabolism within the PSA gray zone of 4–10 ng/ml in PCa patients compared with BPH patients was firstly explored by LC-MS/MS analysis. Significant differences in glycerophospholipids, glycerolipid, and arachidonic acid metabolisms were observed between the two groups. Consistent with our findings, previous studies have reported the same dysregulation of glycerophospholipids, glycerolipid, and arachidonic acid metabolisms in various cancers (30), including lung cancer (32), breast cancer (33), colorectal cancer (34), ovarian cancer (35), pancreatic cancer (36), kidney cancer (37), and gastric cancer (13). A total of 18 lipid-related metabolites (including 4-oxoretinol, anandamide, palmitic acid, and glycidyl stearate) was identified using the selection criteria of variable VIP > 2, FC >1.5 or <2/3, and *p* < 0.05. These metabolites were downregulated in PCa patients compared with the BPH control subjects. The significant decrease in lipid profiles in PCa patients can be attributed to increased lipid utilization by neoplastic cells for membrane biogenesis (a characteristic of carcinogenesis), and increased requirement of phospholipids during fast cell proliferation (13, 38). Notably, dysregulated lipid metabolism can be caused by physiological factors of PCa subjects, such as hormone generation and expression of oncogenes (39). Androgens induce transcription of enzymes to activate lipid metabolism in PCa by inducing SREBP1 transcription factor (40, 41). Additionally, lipid metabolism-related oncogenes such as P53, fatty acid transporting genes (FABP), lipogenic genes (FAS), and lipolytic genes (ATGL) are implicated in regulation of metabolic processes, including glycolysis and lipogenesis (42, 43). However, the metabolic profile differences in PCa with various gene polymorphisms were not explored in the current study owing to lack of sufficient patient information. Therefore, further studies should focus on the metabolic profiles of different gene expressions.

Lipid metabolism with elevated cholesterol synthesis and steroid genesis favor development of PCa disease compared with other solid tumors (40, 44). In the current study, significantly higher levels of TC, LDL-C, and HDL-C were observed in PCa patients compared with those in HC and BPH controls. All the 18 newly identified metabolites were significantly negatively correlated with TC, LDL-C, and Apo-B levels in PCa patients, whereas only a few metabolites were negatively correlated with HDL-C and Apo-A levels. Analysis showed no correlation between TG level and the 18 candidate biomarkers, indicating that the metabolites were not implicated in TG metabolism. We speculated that TG present in circulation may not exert significant effects on the pathogenesis and progression of PCa, but additional research is warranted. Lipids are currently used as effective biomarkers for several diseases in various clinical applications (13). In the present study, all the 18 metabolites hold the promise as alternative diagnostic tools for discrimination of PCa from BPH at the gray zone of PSA 4–10 ng/ml (all AUC > 0.80).

Potential application of metabolomics in PCa has attracted great attention following an unbiased metabolomics analysis by Sreekumar et al. The researchers found that metabolites including sarcosine, uracil, kynurenine, glycerol-3-phosphate, leucine, and proline exhibit high levels during PCa progression from benign adjacent prostate (n = 16) to localized PCa (n = 12) to metastatic PCa (n = 14) (45). Sarcosine displayed the most significant differences, with elevated levels in 79% of metastatic tumor tissues and 42% of localized tumor tissues and absent in benign specimens. In addition, McDunn et al. analyzed metabolites in two cohorts with 331 PCa tissue samples and 178 benign tissue (matched to 178 of the PCa specimens) samples using GC-MS and LC-MS/MS methods (46). Almost all of the significantly altered metabolites reported by Sreekumar et al. were observed in the study by McDunn et al., including high levels of the progression-associated metabolites glycerol-3-phosphate, kynurenine, proline, threonine, and uracil in PCa tissue compared with the levels in benign tissue. But under the findings of McDunn et al., the sarcosine level was only significantly raised in tissue samples with a Gleason score of 8 or higher. Metabolomics studies have further reported elevated alterations of sarcosine levels in urine/serum/plasma samples of PCa patients (18, 19). In summary, these findings suggest that sarcosine is a potential effective biomarker for PCa and can be used for differentiation of high-grade PCa from low-grade PCa. In addition to the in-depth study of sarcosine, dysregulation of lipid metabolism in PCa is also extensively explored as a promising approach for assessing disease progression and outcome. For example, Lin et al. studied the correlation between the plasma lipid profiles and clinical outcomes in 96 patients with castrate-refractory PCa. A three-lipid signature comprising ceramide d18:1/24:1, sphingomyelin d18:2/16:0, and phosphatidylcholine 16:0/16:0 was established. Eleven subjects from the validation cohort (63 patients) exhibited the three-lipid signature and had a significant shorter median overall survival (11.3 months) than did patients who did not exhibit the signature (21.4 months) (47). These findings show significant progress in use of lipid metabolites for PCa prognosis. Moreover, fatty acids are catabolized by fatty acid oxidation, which is an essential energy source for abnormal cell proliferation (19); thereby, dysregulation of fatty acid metabolism pathway is vital in the development of cancers. Furthermore, alterations in energetic metabolism are common in tumors. Increased serum glucose level is correlated with an increased risk of PCa recurrence after radical resection or radiation therapy (19). Despite the promising data and the numerous urinary and serum biomarkers under investigation for PCa diagnosis, many critical issues should be covered in the near future to implement their use in clinical practice.

In summary, the present study provides a novel insight into PCa diagnosis in the gray zone of 4~10 ng/ml. These newly found biomarkers would manage PCa patients by guiding biopsy and improving on the false-positive rate of PSA (48). Furthermore, combining blood biomarkers with multiparametric magnetic resonance imaging (mpMRI) would be of particular value for PCa management in clinic (49). However, the study had some limitations. Firstly, the study cohort size was small and lacked an external validation cohort; therefore, the models may have overfitting bias. Secondly, one-time measurement of metabolites and processing delays in sample preparation may increase risk of measurement error, thus affecting associations of metabolites with PCa risk. Thirdly, a multi-omics analysis integrating data on genes, proteins, and metabolites should be performed to gain deeper insights into the potential mechanisms of metabolites in modulating occurrence and progression of PCa.

## Conclusion

Analysis of serum samples through LC-MS/MS-based metabolomics successfully discriminated patients and controls with BPH at the gray zone of PSA 4~10 ng/ml. Significantly enriched pathways including glycerophospholipid, glycerolipid, and arachidonic acid metabolisms were observed in PCa patients. Eighteen novel lipid or lipid-like metabolites were identified as potential biomarkers for distinguishing PCa patients from BPH cases in the PSA gray zone of 4–10 ng/ml. These findings provide a basis for addressing the current medical challenge of poor specificity of serum PSA for diagnosis of PCa.

## Data Availability Statement

The datasets during and/or analyzed during the current study are available from the corresponding author on reasonable request.

## Ethics Statement

The study was approved by the Medical Ethics Committee of Mianyang Central Hospital (approval no. P2020040). All patients provided written informed consent to participate.

## Author Contributions

All authors contributed to the study conception and design and take responsibility for the integrity of the data and the accuracy of the data analysis. Data collection was performed by BX, LG, and YZ; and analysis was performed by XC and YC. Material preparation and the first draft of the manuscript was written by JF and LY; and all authors commented on previous versions of the manuscript. All authors contributed to the article and approved the submitted version.

## Funding

This work was financially supported by the Sichuan Health and Health Committee Support Program (20PJ255), Incubation Project of Mianyang Central Hospital (2019FH01 and 2019YJ22), and CSCO-qilu cancer scientific research fund (NO.Y-QL202101-0125).

## Conflict of Interest

Author XC was employed by SCIEX Analytical Instrument Trading Co.

The remaining authors declare that the research was conducted in the absence of any commercial or financial relationships that could be construed as a potential conflict of interest.

## Publisher’s Note

All claims expressed in this article are solely those of the authors and do not necessarily represent those of their affiliated organizations, or those of the publisher, the editors and the reviewers. Any product that may be evaluated in this article, or claim that may be made by its manufacturer, is not guaranteed or endorsed by the publisher.
