# Supplementary material for: Metabolomics Profiling Discriminates Prostate Cancer From Benign Prostatic Hyperplasia Within the Prostate-Specific Antigen Gray Zone
Source: Front Oncol. 2021 Oct 15;11:730638. doi: 10.3389/fonc.2021.730638 (PMC8554118; doi:10.3389/fonc.2021.730638)
Supplement: Supplementary file 1 [file DataSheet_1.docx]

**Supplementary material-Tables**

**Table S1 Significantly altered metabolic pathways and metabolites between PCa and BPH groups**

| **Pathway name** | **Hits** | **Metabolites** |
| --- | --- | --- |
| Glycerophospholipid metabolism | 6 | Phosphatidylethanolamine (PE)  Phosphatidylcholine (PC)  1-Acyl-sn-glycero-3-phosphocholine  Phosphatidic acid (PA)  Phosphatidylserine (PS)  Phosphatidylglycerol (PG) |
| Glycerolipid metabolism | 3 | Phosphatidate  1-Acylglycerol  D-Galactosyldiacylglycerol |
| Arachidonic acid metabolism | 2 | Arachidonic acid  Phosphatidylcholine |
| Nicotinate and nicotinamide metabolism | 1 | Nicotinate D-ribonucleotide |
| Biotin metabolism | 1 | Biocytin |

^1^ Hits, the number of differential metabolites matching the pathway.

**Table S2 Significantly altered metabolic pathways and metabolites between PCa and HC groups**

| **Pathway name** | **KEGG.id** | **-log(P)** | **Impact** | **Hits** | **Metabolites** |
| --- | --- | --- | --- | --- | --- |
| Glycerophospholipid metabolism | hsa00564 | 5.25 | 0.39 | 6 | Phosphatidylethanolamine (PE)  Phosphatidylcholine (PC)  1-Acyl-sn-glycero-3-phosphocholine  Phosphatidic acid (PA)  1-Acyl-sn-glycero-3-phosphoethanolamine  Phosphatidylglycerol (PG) |
| Glycerolipid metabolism | hsa00561 | 3.36 | 0.22 | 3 | Phosphatidate  1-Acylglycerol  D-Galactosyldiacylglycerol |
| Arachidonic acid metabolism | hsa00590 | 0.68 | 0.31 | 2 | Arachidonic acid  Phosphatidylcholine |
| Nicotinate and nicotinamidemetabolism | hsa00760 | 0.67 | 0.19 | 1 | Nicotinate D-ribonucleotide |
| Biotin metabolism | hsa00780 | 0.97 | 0.15 | 1 | Biocytin |

^1^ Impact, impact value of metabolic pathway determined by topology analysis; ^2^ Hits, the number of differential metabolites matching the pathway.

**Supplementary material-Figures**


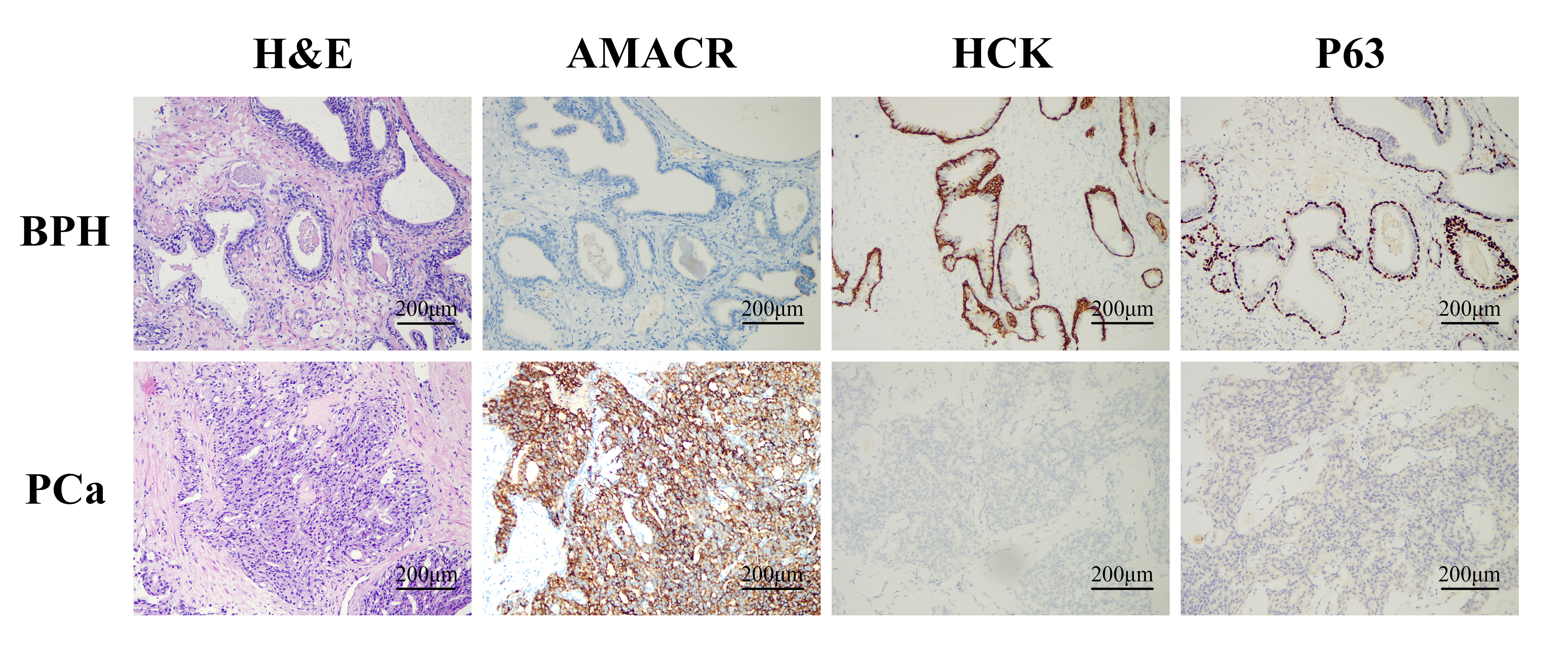


Fig.S1 Represent pathological section staining images of prostatic tissues in BPH and PCa patients. Overexpression of AMACR in combination with absence of basal cell markers HCK and P63 is typical of classic prostatic adenocarcinoma. H&E, hematoxylin and eosin; AMACR, alpha-methylacyl coenzyme A racemase; HCK, high molecular weight cytokeratin.


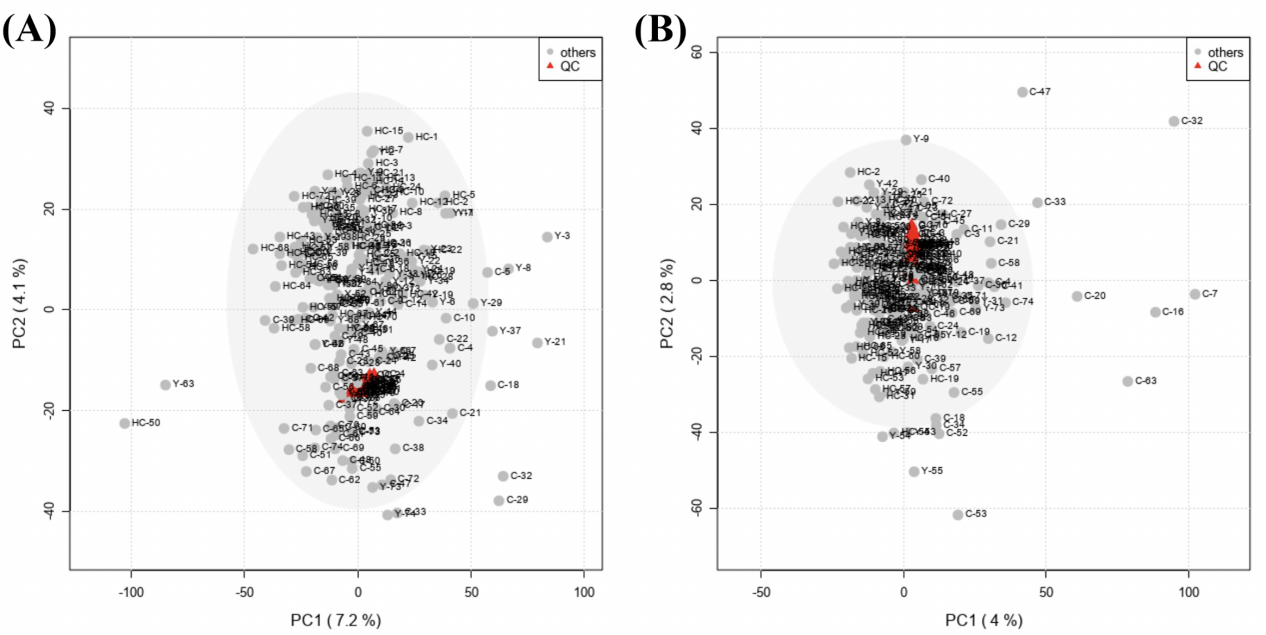


Fig.S2 PCA score plots of QC samples In positive (A) and negative (B) ion modes. Results revealed the analysis had satisfactory stability and repeatability of metabolomic datasets.


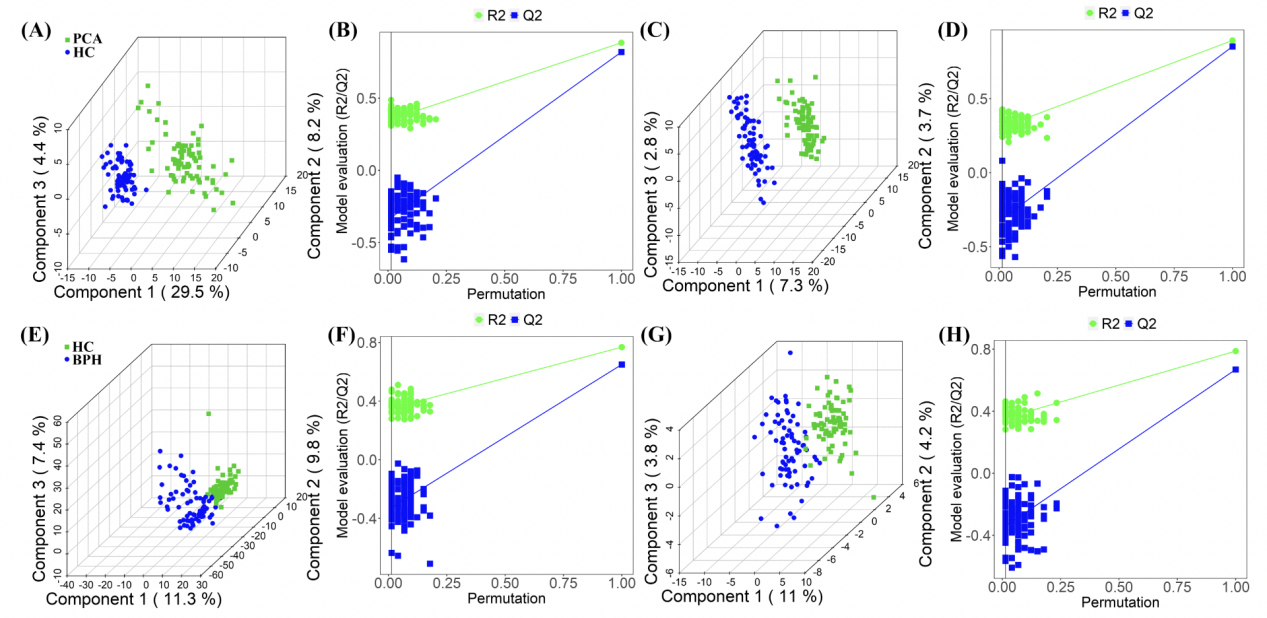


Fig.S3 Plots of PLS-DA score and permutation test between HC group and PCa/BPH groups. PLS-DA score plot in positive (A) and negative (C) ion modes between HC group and PCa group. PLS-DA permutation test plot in positive (B) and negative (D) ion modes between HC group and PCa group. PLS-DA score plot in positive (E) and negative (G) ion modes between HC group and BPH group. PLS-DA permutation test plot in positive (F) and negative (H) ion modes between HC group and BPH group.
